# Partial maximum correntropy regression for robust electrocorticography decoding

**DOI:** 10.3389/fnins.2023.1213035

**Published:** 2023-06-30

**Authors:** Yuanhao Li, Badong Chen, Gang Wang, Natsue Yoshimura, Yasuharu Koike

**Affiliations:** ^1^Institute of Innovative Research, Tokyo Institute of Technology, Yokohama, Japan; ^2^Institute of Artificial Intelligence and Robotics, Xi'an Jiaotong University, Xi'an, China; ^3^Key Laboratory of Biomedical Information Engineering of Ministry of Education, Xi'an Jiaotong University, Xi'an, China; ^4^School of Computing, Tokyo Institute of Technology, Yokohama, Japan

**Keywords:** brain-computer interface, partial least square regression, maximum correntropy, robustness, electrocorticography decoding

## Abstract

The *Partial Least Square Regression* (PLSR) method has shown admirable competence for predicting continuous variables from inter-correlated electrocorticography signals in the brain-computer interface. However, PLSR is essentially formulated with the least square criterion, thus, being considerably prone to the performance deterioration caused by the brain recording noises. To address this problem, this study aims to propose a new robust variant for PLSR. To this end, the maximum correntropy criterion (MCC) is utilized to propose a new robust implementation of PLSR, called *Partial Maximum Correntropy Regression* (PMCR). The half-quadratic optimization is utilized to calculate the robust projectors for the dimensionality reduction, and the regression coefficients are optimized by a fixed-point optimization method. The proposed PMCR is evaluated with a synthetic example and a public electrocorticography dataset under three performance indicators. For the synthetic example, PMCR realized better prediction results compared with the other existing methods. PMCR could also abstract valid information with a limited number of decomposition factors in a noisy regression scenario. For the electrocorticography dataset, PMCR achieved superior decoding performance in most cases, and also realized the minimal neurophysiological pattern deterioration with the interference of the noises. The experimental results demonstrate that, the proposed PMCR could outperform the existing methods in a noisy, inter-correlated, and high-dimensional decoding task. PMCR could alleviate the performance degradation caused by the adverse noises and ameliorate the electrocorticography decoding robustness for the brain-computer interface.

## 1. Introduction

Brain-computer interface (BCI) has been conceived as a promising technology that translates cerebral recordings generated by cortical neurons into appropriate commands for controlling neuroprosthetic devices (Wolpaw et al., 2002). The capability of BCI for repairing or reproducing sensory-motor functions has been increasingly intensified by recent scientific and technological advances (Donoghue, 2002; Mussa-Ivaldi and Miller, 2003; Lebedev and Nicolelis, 2006). The non-invasive recordings, especially electroencephalogram (EEG) and magnetoencephalogram (MEG), are widely exploited to structure BCI systems due to their ease of use and satisfactory temporal resolution, whereas the non-invasive BCI systems could be limited in their capabilities and customarily require considerable training (Amiri et al., 2013). Invasive single-unit activities and local field potentials commonly provide better decoding performance, which suffer pessimistic long-term stability, however, due to capriciousness in the recorded neuronal-ensembles (Chestek et al., 2007). A sophisticated alternative which exhibits higher signal amplitudes than EEG while presents superior long-term stability compared with invasive modalities, is the semi-invasive electrocorticography (ECoG) (Buzsáki et al., 2012). Numerous studies in recent years have investigated the potentials of ECoG signal for decoding motions (Levine et al., 2000; Leuthardt et al., 2004; Chin et al., 2007; Pistohl et al., 2008; Ball et al., 2009b; Chao et al., 2010; Shimoda et al., 2012). The serviceability of ECoG signal for online practice have also been demonstrated in Leuthardt et al. (2004, 2006), Schalk et al. (2008).

To accomplish the inter-correlated and potentially high-dimensional ECoG decoding tasks, the *partial least square regression* (PLSR) algorithm has been widely utilized to predict continuous variables from ECoG signals as well as various improved versions in the last decade (Chao et al., 2010; Eliseyev et al., 2011, 2012, 2017; Shimoda et al., 2012; Zhao et al., 2012, 2013; Eliseyev and Aksenova, 2016; Foodeh et al., 2020). Chao et al. (2010) successfully predicted the three-dimensional continuous hand trajectories of two monkeys during asynchronous food-reaching tasks from time-frequency features of subdural ECoG signals by PLSR algorithm. They further showed the admirable prediction capability of PLSR in an epidural ECoG study (Shimoda et al., 2012). Recently, different strategies have been investigated to improve the decoding performance of PLSR. For instance, multi-way PLSR algorithms have been proposed as a generalization for tensor analysis in the ECoG decoding tasks (Bro, 1996; Shimoda et al., 2012; Zhao et al., 2013; Eliseyev et al., 2017). Moreover, regularization technique has been used to penalize the objective function with an extra regularization term to achieve desirable prediction (Eliseyev et al., 2012; Eliseyev and Aksenova, 2016; Foodeh et al., 2020). Although the PLSR algorithm was initially developed for econometrics and chemometrics (Wold, 1966), it has emerged as a popular method for neural imaging and decoding (Krishnan et al., 2011; Zhao et al., 2014).

PLSR solves a regression problem primarily with dimensionality reduction on both explanatory matrix (input) and response matrix (output), in which the dimensionality-reduced samples (commonly called as *latent variables*) for respective sets exhibit maximal correlation, thus structuring association from input variables to output variables. Nevertheless, the conventional PLSR and most existing variants are in essence formulated by the least square criterion, which assigns superfluous importance to the deviated noises. On the other hand, although ECoG signal usually exhibits a relatively higher signal-to-noise ratio (SNR) than the non-invasive EEG recording, previous studies have revealed that ECoG is also prone to be contaminated by physiological artifacts with pronounced amplitudes (Otsubo et al., 2008; Ball et al., 2009a). As a result, PLSR could be incompetent for noisy ECoG decoding tasks due to subnormal robustness.

The present study aims to propose a novel robust version for PLSR through introducing the *maximum correntropy criterion* (MCC) to replace the conventional least square criterion, which was proposed in the *information theoretic learning* (ITL) (Principe, 2010), and has achieved the state-of-the-art robust approaches in different tasks, including regression (Liu et al., 2007; Chen and Pŕıncipe, 2012; Feng et al., 2015), classification (Singh et al., 2014; Ren and Yang, 2018), principal component analysis (He et al., 2011), and feature extraction (Dong et al., 2017). Recently, a rudimentary implementation of the MCC in the PLSR algorithm has been investigated in Mou et al. (2018), where MCC was employed in the process of dimensionality reduction. However, the proposed algorithm in Mou et al. (2018) may be limited in some respects. First, except for the MCC-based dimensionality reduction, it remains acquiring the regression relations under the least square criterion. Second, it only considers the dimensionality reduction for the explanatory matrix. Consequently, one has to calculate the regression coefficients separately for each dimension of the response matrix, which means it could be inadequate for multivariate response prediction.

By comparison, the present study aims to realize a more comprehensive implementation of the MCC framework in PLSR. The main contributions of this study are summarized as follows.

1. We reformulate PLSR thoroughly with the MCC framework, that not only the dimensionality reduction, but also the regression relations between the different variables are established by the MCC framework.
2. Both the explanatory matrix (input) and the response matrix (output) are treated with MCC-based dimensionality reduction. As a result, the proposed algorithm is adequate for multivariate response prediction.
3. We utilize Gaussian kernel functions with individual kernel bandwidths for different reconstruction errors and prediction errors. In addition, each kernel bandwidth value could be calculated from the corresponding set of errors directly.

The remainder of this paper is organized as follows. Section 2 introduces the conventional PLSR method as well as the regularized versions. Section 3 gives a brief introduction about MCC and the rudimentary MCC-based PLSR algorithm. Section 4 presents the reformulation of PLSR with the MCC framework, proposing the *partial maximum correntropy regression* (PMCR) algorithm. Section 5 evaluates the proposed method on synthetic and real ECoG datasets, respectively. Some discussions about the proposed method are given in Section 6. Finally, this paper is concluded in Section 7. To facilitate the presentation of this paper, the main notations are listed in Table 1.

**Table 1:** Main notations.

**Notation**	**Description**
*L*	Number of observations/samples
*N*	Dimension of explanatory matrix (input)
*M*	Dimension of response matrix (output)
*S*	Optimal number of decomposition factors
*s*	Current index of decomposition factor
**X**	Original explanatory matrix (input)
**Y**	Original response matrix (output)
$\hat{\text{Y}}$	Prediction of response matrix
**X**_*s*_	Residual matrix of **X** in *s*-th factor
**Y**_*s*_	Residual matrix of **Y** in *s*-th factor
${\text{x}}_{s}^{l}$	*l*-th observation in **X**_*s*_
${\text{y}}_{s}^{l}$	*l*-th observation in **Y**_*s*_
**w**_*s*_	Dimensionality-reduction projector for **X**_*s*_
**c**_*s*_	Dimensionality-reduction projector for **Y**_*s*_
**t**_*s*_	Input latent variables in *s*-th factor
**u**_*s*_	Output latent variables in *s*-th factor
**p**_*s*_	Loading vector in *s*-th factor
*b*_*s*_	Regression coefficient between **t**_*s*_ and **u**_*s*_
*g*_σ_(·)	Gaussian kernel function with kernel bandwidth σ

## 2. Partial least square regression

### 2.1. Conventional PLSR

Consider the data set with the explanatory matrix **X**∈ℝ^*L*×*N*^ and the response matrix **Y**∈ℝ^*L*×*M*^, in which *N* and *M* denote the respective numbers of dimension, while *L* is the number of observations. PLSR is an iterative regression method which implements dimensionality reduction and decomposition on explanatory and response matrices simultaneously for *S* iterations, so that they could be expressed by


(1) $$\begin{array}{l} \text{X}=\text{T}{\text{P}}^{T},\text{Y}=\text{T}\text{B}{\text{C}}^{T} \end{array}$$

where $\text{T}=\left[{\text{t}}_{1},..,{\text{t}}_{S}\right]\in {\mathbb{R}}^{L\times S}$ and $\text{P}=\left[{\text{p}}_{1},..,{\text{p}}_{S}\right]\in {\mathbb{R}}^{N\times S}$ are the latent variables and loading vectors for **X**, respectively. $\text{C}=\left[{\text{c}}_{1},..,{\text{c}}_{S}\right]\in {\mathbb{R}}^{M\times S}$ is the loading vectors of **Y**, and $\text{B}=\text{diag}\left(b_{1},..,b_{S}\right)\in {\mathbb{R}}^{S\times S}$ is a diagonal matrix. For dimensionality reduction, in the *s*-th iteration with residual matrices **X**_*s*_ and **Y**_*s*_, the covariance between the latent variables **t**_*s*_ = **X**_*s*_**w**_*s*_ and **u**_*s*_ = **Y**_*s*_**c**_*s*_ are maximized by


(2) $$\begin{array}{l} \underset{∥{\text{w}}_{s}{∥}_{2}=∥{\text{c}}_{s}{∥}_{2}=1}{\max}{\text{t}}_{s}^{T}{\text{u}}_{s}={\text{w}}_{s}^{T}{\text{X}}_{s}^{T}{\text{Y}}_{s}{\text{c}}_{s} \end{array}$$

in which ${\text{w}}_{s}\in {\mathbb{R}}^{N}$ and ${\text{c}}_{s}\in {\mathbb{R}}^{M}$ are utilized for dimensionality reduction on **X**_*s*_ and **Y**_*s*_, respectively. **u**_*s*_ is the latent variable for **Y**_*s*_. ∥·∥_2_ denotes the *L*_2_-norm. After obtaining the latent variables **t**_*s*_ and **u**_*s*_, the loading vector **p**_*s*_ and the relation from **t**_*s*_ to **u**_*s*_ with the scalar *b*_*s*_ are founded by the least square criterion


(3) $$\begin{array}{l} \underset{{\text{p}}_{s}}{\min}∥{\text{X}}_{s}-{\text{t}}_{s}{\text{p}}_{s}^{T}{∥}_{2}^{2}\Rightarrow {\text{p}}_{s}={\text{X}}_{s}^{T}{\text{t}}_{s}/\left({\text{t}}_{s}^{T}{\text{t}}_{s}\right) \end{array}$$

(4) $$\begin{array}{l} \underset{b_{s}}{\min}∥{\text{u}}_{s}-{\text{t}}_{s}b_{s}{∥}_{2}^{2}\Rightarrow b_{s}={\text{u}}_{s}^{T}{\text{t}}_{s}/\left({\text{t}}_{s}^{T}{\text{t}}_{s}\right) \end{array}$$

The residual matrices are updated by ${\text{X}}_{s+1}={\text{X}}_{s}-{\text{t}}_{s}{\text{p}}_{s}^{T}$ and ${\text{Y}}_{s+1}={\text{Y}}_{s}-b_{s}{\text{t}}_{s}{\text{c}}_{s}^{T}$. *S* is usually selected by cross validation. Eventually, the prediction from **X** to **Y** is structured by


(5) $$\begin{array}{l} \hat{\text{Y}}=\text{X}\text{H} \end{array}$$

where **H** = **P**^*T*+^**BC**^*T*^∈ℝ^*N*×*M*^, and **P**^*T*+^ is the pseudo-inverse of **P**^*T*^. $\hat{\text{Y}}$ denotes the prediction for **Y**.

Maximizing the covariance between latent variables Eq. (2) could be rewritten as (Barker and Rayens, 2003).


(6) $$\begin{array}{l} \underset{∥{\text{w}}_{s}{∥}_{2}=∥{\text{c}}_{s}{∥}_{2}=1}{\min}\overset{L}{\underset{l=1}{\sum }}\left(\begin{array}{c} ∥{\text{x}}_{s}^{l}-{\text{x}}_{s}^{l}{\text{w}}_{s}{\text{w}}_{s}^{T}{∥}^{2}\\ +∥{\text{y}}_{s}^{l}-{\text{y}}_{s}^{l}{\text{c}}_{s}{\text{c}}_{s}^{T}{∥}^{2}\\ +∥{\text{x}}_{s}^{l}{\text{w}}_{s}-{\text{y}}_{s}^{l}{\text{c}}_{s}{∥}^{2} \end{array}\right) \end{array}$$

where ${\text{x}}_{s}^{l}$ and ${\text{y}}_{s}^{l}$ denote the *l*-th samples in **X**_*s*_ and **Y**_*s*_, respectively. One can observe that, PLSR employs the least square criterion not only to obtain the regression relations in Eqs. (3, 4), but for the projectors **w**_*s*_ and **c**_*s*_ as well. In Eq. (6), the first and second terms are the reconstruction errors for input and output, respectively. The third term denotes the prediction error for the *l*-th latent variables. Since each step for PLSR is based on the least square criterion, the prediction from input to output could be seriously deteriorated by noises.

### 2.2. Regularized PLSR

Regularization technique has been popularly employed to ameliorate the decoding performance of the PLSR algorithm. For example, *L*_1_-regularization on the projectors was employed so as to acquire sparse projectors, conducting the feature selection simultaneously (Eliseyev et al., 2012). The authors further extended their study in Eliseyev and Aksenova (2016), in which Sobolev-norm and polynomial penalization were introduced into PLSR algorithm to strengthen the smoothness of the predicted response. Recently, the state-of-the-art regularized PLSR was proposed by utilizing *L*_2_-regularization to find the regression relation between the latent variables **t**_*s*_ and **u**_*s*_, so as to reduce the over-fitting risk of each latent variable on the desired response (Foodeh et al., 2020). In particular, for each decomposition factor, the scalar *b*_*s*_ is acquired with an individual regularization parameter λ_*s*_ as


(7) $$\begin{array}{l} \underset{b_{s}}{\min}∥{\text{u}}_{s}-{\text{t}}_{s}b_{s}{∥}_{2}^{2}+\lambda_{s}b_{s}^{2}\Rightarrow b_{s}={\text{u}}_{s}^{T}{\text{t}}_{s}/\left({\text{t}}_{s}^{T}{\text{t}}_{s}+\lambda_{s}\right) \end{array}$$

Experimental results in Foodeh et al. (2020) showed that, the regularization technique in Eq. (7) can achieve better ECoG decoding performance than regularizing the projectors.

Nevertheless, the regularized PLSR variants remain formulated based on the non-robust least square criterion, as a result, being still prone to suffering the performance deterioration caused by the adverse noises.

## 3. Maximum correntropy criterion

### 3.1. Maximum correntropy criterion

The correntropy concept was developed in the field of ITL as a generalized correlation function of random processes (Santamaŕıa et al., 2006), which measures the similarity and interaction between two vectors in a kernel space. Correntropy associates with the information potential of quadratic Renyi's entropy (Liu et al., 2007), where the data's probability density function (PDF) is estimated by the Parzen's window method (Parzen, 1962; Silverman, 1986). The correntropy which evaluates the similarity between two arbitrary variables *A* and *B*, is defined by


(8) $$\begin{array}{l} V\left(A,B\right)=E\left[k\left(A-B\right)\right] \end{array}$$

in which *k*(·) is a kernel function satisfying the Mercer's theory and *E*[·] is the expectation operator. In the practical application, one calculates the correntropy with *L* observations by the following empirical estimation


(9) $$\begin{array}{l} \hat{V}\left(A,B\right)=\frac{1}{L}\overset{L}{\underset{l=1}{\sum }}k\left(a_{l}-b_{l}\right) \end{array}$$

where the Gaussian kernel function $g_{\sigma }\left(x\right)≜\exp\left(-x^{2}/2\sigma^{2}\right)$ with kernel bandwidth σ is widely used for the kernel function *k*(·), thus leading to


(10) $$\begin{array}{l} \hat{V}\left(A,B\right)=\frac{1}{L}\overset{L}{\underset{l=1}{\sum }}g_{\sigma }\left(a_{l}-b_{l}\right)=\frac{1}{L}\overset{L}{\underset{l=1}{\sum }}\exp\left(-\frac{{\left(a_{l}-b_{l}\right)}^{2}}{2\sigma^{2}}\right) \end{array}$$

Maximizing the correntropy Eq. (10), called as the *maximum correntropy criterion* (MCC), exhibits numerous advantages. Correntropy is essentially a local similarity measure, which is chiefly determined along *A* = *B*, i.e. zero-value error. Consequently, the effect of large error caused by adverse noise is alleviated, leading to superior robustness. Additionally, correntropy could extract sufficient information from observations, since it considers all the even moments of errors (Liu et al., 2007). It also relates closely to the *m*-estimation, which can be regarded as a robust formulation of Welsch *m*-estimator (Huber, 2004).

### 3.2. MCC-PLSR

Recently, a rudimentary MCC-based PLSR variant has been investigated in Mou et al. (2018), named as MCC-PLSR. For a univariate output, according to Mou et al. (2018), the dimensionality reduction Eq. (2) could be rewritten as


(11) $$\begin{array}{l} \underset{∥{\text{w}}_{s}{∥}_{2}=1}{\max}{\text{w}}_{s}^{T}{\text{X}}_{s}^{T}{\text{Y}}_{s}{\text{Y}}_{s}^{T}{\text{X}}_{s}{\text{w}}_{s} \end{array}$$

which aims to maximize the quadratic covariance. Mou et al. (2018) utilized a similar proposition as in the MCC-based principal component analysis (He et al., 2011), proposing the following objective function


(12) $$\begin{array}{l} \underset{∥{\text{w}}_{s}{∥}_{2}=1}{\max}\overset{L}{\underset{l=1}{\sum }}g_{\sigma }\left(\sqrt{{\text{y}}_{s}^{lT}{\text{x}}_{s}^{l}{\text{x}}_{s}^{lT}{\text{y}}_{s}^{l}-{\text{y}}_{s}^{lT}{\text{x}}_{s}^{l}{\text{w}}_{s}{\text{w}}_{s}^{T}{\text{x}}_{s}^{lT}{\text{y}}_{s}^{l}}\right) \end{array}$$

from which one can calculate the robust projector **w**_*s*_. Then, one obtains the latent variables by **t**_*s*_ = **X**_*s*_**w**_*s*_, and acquires other model parameters similarly as in Eqs. (3-5).

Despite the robust implementation of the projector **w**_*s*_ in Eq. (12), the above-described MCC-PLSR algorithm could be inadequate for the following reasons. First, except for the calculation of **w**_*s*_, the other model parameters are still acquired under the least square criterion. Second, dimensionality reduction is not considered for the output matrix. As a result, the prediction performance for multivariate response could be limited. In addition, MCC-PLSR is prone to suffering excessive computation time, since one has to obtain the prediction model $\hat{\text{Y}}=\text{X}\text{H}$ for each dimension of the response matrix separately.

## 4. Partial maximum correntropy regression

In this section, we present a comprehensive reformulation of PLSR with the MCC framework. Compared with the existing MCC-PLSR, our proposed method aims to acquire each model parameter under the MCC. In addition, the generalization for multivariate response prediction is taken into account in this study. The detailed mathematical derivations of the proposed method are given as follows, in which the subscript *s* denoting the *s*-th decomposition factor is omitted for the purpose of simplicity.

Substituting the least quadratic reconstruction errors and prediction errors in the conventional PLSR Eq. (6) with the maximum correntropy yields


(13) $$\begin{array}{l} \underset{∥\text{w}{∥}_{2}=∥\text{c}{∥}_{2}=1}{\max}\overset{L}{\underset{l=1}{\sum }}\left(\begin{array}{c} g_{\sigma_{x}}\left({\text{x}}^{l}-{\text{x}}^{l}\text{w}{\text{w}}^{T}\right)\\ +g_{\sigma_{y}}\left({\text{y}}^{l}-{\text{y}}^{l}\text{c}{\text{c}}^{T}\right)\\ +g_{\sigma_{r}}\left({\text{x}}^{l}\text{w}-{\text{y}}^{l}\text{c}\right) \end{array}\right) \end{array}$$

where σ_*x*_, σ_*y*_, and σ_*r*_ denote the Gaussian kernel bandwidths for **X**-reconstruction errors, **Y**-reconstruction errors, and the prediction errors, respectively.

Then, one can transform the vectors (**x**^*l*^−**x**^*l*^**ww**^*T*^) and (**y**^*l*^−**y**^*l*^**cc**^*T*^) into scalars, provided that the two projectors **w** and **c** are unit-length vectors, i.e. **w**^*T*^**w** = **c**^*T*^**c** = 1,


(14) $$\begin{array}{l} \begin{array}{cc} \sqrt{∥{\text{x}}^{l}-{\text{x}}^{l}\text{w}{\text{w}}^{T}{∥}^{2}} & =\sqrt{{\text{x}}^{l}{\text{x}}^{lT}-{\text{x}}^{l}\text{w}{\text{w}}^{T}{\text{x}}^{lT}}\\ \sqrt{∥{\text{y}}^{l}-{\text{y}}^{l}\text{c}{\text{c}}^{T}{∥}^{2}} & =\sqrt{{\text{y}}^{l}{\text{y}}^{lT}-{\text{y}}^{l}\text{c}{\text{c}}^{T}{\text{y}}^{lT}} \end{array} \end{array}$$

Subsequently, one obtains the following optimization problem to acquire the projectors


(15) $$\begin{array}{l} \underset{∥\text{w}{∥}_{2}=∥\text{c}{∥}_{2}=1}{\max}\overset{L}{\underset{l=1}{\sum }}\left(\begin{array}{c} g_{\sigma_{x}}\left(\sqrt{{\text{x}}^{l}{\text{x}}^{lT}-{\text{x}}^{l}\text{w}{\text{w}}^{T}{\text{x}}^{lT}}\right)\\ +g_{\sigma_{y}}\left(\sqrt{{\text{y}}^{l}{\text{y}}^{lT}-{\text{y}}^{l}\text{c}{\text{c}}^{T}{\text{y}}^{lT}}\right)\\ +g_{\sigma_{r}}\left({\text{x}}^{l}\text{w}-{\text{y}}^{l}\text{c}\right) \end{array}\right) \end{array}$$

After obtaining **w** and **c**, one could calculate the latent variables as in the conventional PLSR by **t** = **Xw** and **u** = **Yc**. We then calculate the loading vector **p** and the regression coefficient *b* under the MCC by


(16) $$\begin{array}{l} \underset{\text{p}}{\max}\overset{L}{\underset{l=1}{\sum }}g_{\sigma_{p}}\left({\text{x}}^{l}-{\text{t}}^{l}{\text{p}}^{T}\right) \end{array}$$

(17) $$\begin{array}{l} \underset{b}{\max}\overset{L}{\underset{l=1}{\sum }}g_{\sigma_{b}}\left({\text{u}}^{l}-{\text{t}}^{l}b\right) \end{array}$$

in which **t**^*l*^ and **u**^*l*^ denote the *l*-th elements for the latent variables **t** and **u**, respectively. σ_*p*_ and σ_*b*_ denote the corresponding Gaussian kernel bandwidths. The residual matrices are then updated similarly as PLSR.

One repeats such procedures for the optimal number of factors and collects the acquired vectors from each iteration to organize the matrices **T**, **P**, **B**, and **C**, as in the original PLSR. Ultimately, the predicted response $\hat{\text{Y}}$ can be obtained from **X** by the regression relationship Eq. (5). The above-mentioned PLSR variant which is comprehensively reformulated based on the MCC, is named as *partial maximum correntropy regression* (PMCR). In what follows, we discuss in detail about the optimization, convergence analysis, and determination of hyper-parameters with regard to the proposed PMCR algorithm.

### 4.1. Optimization

Three optimization problems Eqs. (15, 16, 17) need to be addressed in PMCR. We first consider Eq. (15) for the calculation of the projectors **w** and **c**. Based on the *half-quadratic* (HQ) optimization method (Ren and Yang, 2018), Eq. (15) could be rewritten as


(18) $$\begin{array}{l} \underset{\begin{array}{c} ∥\text{w}{∥}_{2}=\\ ∥\text{c}{∥}_{2}=1 \end{array}}{\max}\overset{L}{\underset{l=1}{\sum }}\left(\begin{array}{c} \sup\left\{\alpha_{l}\frac{{\text{x}}^{l}{\text{x}}^{lT}-{\text{x}}^{l}\text{w}{\text{w}}^{T}{\text{x}}^{lT}}{2\sigma_{x}^{2}}-\varphi \left(\alpha_{l}\right)\right\}\\ +\sup\left\{\beta_{l}\frac{{\text{y}}^{l}{\text{y}}^{lT}-{\text{y}}^{l}\text{c}{\text{c}}^{T}{\text{y}}^{lT}}{2\sigma_{y}^{2}}-\varphi \left(\beta_{l}\right)\right\}\\ +\sup\left\{\gamma_{l}\frac{{\left({\text{x}}^{l}\text{w}-{\text{y}}^{l}\text{c}\right)}^{2}}{2\sigma_{r}^{2}}-\varphi \left(\gamma_{l}\right)\right\} \end{array}\right) \end{array}$$

where φ(·) is a convex conjugated function of *g*(·), and ${\left\{\alpha_{l}\right\}}_{l=1}^{L}$, ${\left\{\beta_{l}\right\}}_{l=1}^{L}$, and ${\left\{\gamma_{l}\right\}}_{l=1}^{L}$ denote three sets of introduced auxiliaries, respectively. Thus, we can conclude that optimizing Eq. (15) is equivalent to updating (α_*l*_, β_*l*_, γ_*l*_) and (**w**, **c**) alternately by


(19) $$\begin{array}{l} \underset{\begin{array}{c} ∥\text{w}{∥}_{2}=\\ ∥\text{c}{∥}_{2}=1,\\ \alpha_{l},\beta_{l},\gamma_{l} \end{array}}{\max}J≜\overset{L}{\underset{l=1}{\sum }}\left(\begin{array}{c} \alpha_{l}\frac{{\text{x}}^{l}{\text{x}}^{lT}-{\text{x}}^{l}\text{w}{\text{w}}^{T}{\text{x}}^{lT}}{2\sigma_{x}^{2}}-\varphi \left(\alpha_{l}\right)\\ +\beta_{l}\frac{{\text{y}}^{l}{\text{y}}^{lT}-{\text{y}}^{l}\text{c}{\text{c}}^{T}{\text{y}}^{lT}}{2\sigma_{y}^{2}}-\varphi \left(\beta_{l}\right)\\ +\gamma_{l}\frac{{\left({\text{x}}^{l}\text{w}-{\text{y}}^{l}\text{c}\right)}^{2}}{2\sigma_{r}^{2}}-\varphi \left(\gamma_{l}\right) \end{array}\right) \end{array}$$

Since the HQ optimization is an iterative process, we denote the *k*-th HQ iteration with the subscript *k*. First, according to the HQ technique (Ren and Yang, 2018), we update the auxiliaries with the current projectors (**w**_*k*_, **c**_*k*_) by


(20) $$\begin{array}{l} \;\alpha_{l,k+1}=-\exp(-\frac{x^{l}x^{lT}-x^{l}w_{k}w_{k}^{T}x^{lT}}{2\sigma_{x}^{2}})\\ \;\beta_{l,k+1}=-\exp(-\frac{y^{l}y^{lT}-y^{l}c_{k}c_{k}^{T}y^{lT}}{2\sigma_{y}^{2}})\;\\ \;\gamma_{l,k+1}=-\exp(-\frac{{\left(x^{l}w_{k}-y^{l}c_{k}\right)}^{2}}{2\sigma_{r}^{2}})\;\\ \;(l=1,..,L)\; \end{array}$$

Then, to optimize the projectors, we rewrite Eq. (19) by collecting the terms of projectors and omitting the auxiliaries as


(21) $$\begin{array}{l} \begin{array}{c} \underset{\begin{array}{c} ∥\text{w}{∥}_{2}=\\ ∥\text{c}{∥}_{2}=1 \end{array}}{\max}J_{p}≜\overset{L}{\underset{l=1}{\sum }}\left(\begin{array}{c} \left(\frac{\gamma_{l}}{2\sigma_{r}^{2}}-\frac{\alpha_{l}}{2\sigma_{x}^{2}}\right){\text{x}}^{l}\text{w}{\text{w}}^{T}{\text{x}}^{lT}\\ +\left(\frac{\gamma_{l}}{2\sigma_{r}^{2}}-\frac{\beta_{l}}{2\sigma_{y}^{2}}\right){\text{y}}^{l}\text{c}{\text{c}}^{T}{\text{y}}^{lT}\\ -\frac{\gamma_{l}}{\sigma_{r}^{2}}{\text{x}}^{l}\text{w}{\text{c}}^{T}{\text{y}}^{lT} \end{array}\right) \end{array} \end{array}$$

which is a quadratic optimization issue constrained by nonlinear conditions. To accomplish Eq. (21), there exist enormous solutions in the literature, such as the sequential quadratic programming (SQP) which has been widely utilized for nonlinear programming problems (Fletcher, 2013).

After one obtains the projectors **w** and **c**, the latent variables are computed by **t** = **Xw** and **u** = **Yc**. Then, Eqs. (16, 17) can be solved by the following iterative fixed-point optimization method with fast convergence (Chen et al., 2015).


(22) $$\begin{array}{l} \text{p}={\text{X}}^{T}\Psi_{\text{p}}\text{t}/\left({\text{t}}^{T}\Psi_{\text{p}}\text{t}\right) \end{array}$$

(23) $$\begin{array}{l} b={\text{u}}^{T}\Psi_{b}\text{t}/\left({\text{t}}^{T}\Psi_{b}\text{t}\right) \end{array}$$

where **Ψ**_**p**_ and **Ψ**_*b*_ are *L*×*L* diagonal matrices with the diagonal elements ${\left(\Psi_{\text{p}}\right)}_{l,l}=g_{\sigma_{p}}\left({\text{x}}^{l}-{\text{t}}^{l}{\text{p}}^{T}\right)$ and ${\left(\Psi_{b}\right)}_{l,l}=g_{\sigma_{b}}\left({\text{u}}^{l}-{\text{t}}^{l}b\right)$, respectively. Since **Ψ**_**p**_ and **Ψ**_*b*_ are dependent on the current solutions **p** and *b*, the updates in Eqs. (22, 23) are fixed-point equations which will require multiple iterations (Chen et al., 2015). The comprehensive procedures for PMCR are summarized in Algorithm 1.

**Algorithm 1:** Partial maximum correntropy regression

1: Input: matrices of explanation **X** and response **Y**; number of factors *S*; a small positive value ς
2: Output: prediction model $\hat{\text{Y}}=\text{X}\text{H}$
3: initialize **X**_1_ = **X** and **Y**_1_ = **Y**;
4: for *s* = 1, 2, .., *S* **do**
5: initialize the projectors by the conventional PLSR;
6: initialize *converged*= FALSE;
7: repeat
8: auxiliary-step: update (α_*l*_, β_*l*_, γ_*l*_) with (20);
9: projector-step: update (**w**_*s*_, **c**_*s*_) with (21);
10: if the difference of the objective function (15) is smaller than ς **then**
11: *converged*= TRUE
12: end **if**
13: until *converged* = = TRUE
14: compute latent variables **t**_*s*_ = **X**_*s*_**w**_*s*_ and **u**_*s*_ = **Y**_*s*_**c**_*s*_;
15: compute **p**_*s*_ and *b*_*s*_ by the fixed-point method (22)(23);
16: update the residual matrices ${\text{X}}_{s+1}={\text{X}}_{s}-{\text{t}}_{s}{\text{p}}_{s}^{T}$ and ${\text{Y}}_{s+1}={\text{Y}}_{s}-b_{s}{\text{t}}_{s}{\text{c}}_{s}^{T}$;
17: end **for**
18: organize the matrices **T** = [**t**_1_, .., **t**_*S*_], **P** = [**p**_1_, .., **p**_*S*_], **B** = diag(*b*_1_, .., *b*_*S*_), and **C** = [**c**_1_, .., **c**_*S*_];
19: compute **H** = **P**^*T*+^**BC**^*T*^

### 4.2. Convergence analysis

For the regression relations **p** and *b*, one could find the detailed convergence analysis in Chen et al. (2015). We mainly consider the convergence of the projectors **w** and **c** in the optimization problem (15). Because correntropy is in nature an *m*-estimator (Liu et al., 2007), the local optimums of Eq. (15) will be close sufficiently to the global optimum, which has been proved in a recent theoretical study (Loh and Wainwright, 2015). Therefore, we prove that Eq. (15) will converge to a local optimum with the HQ optimization method.

Proposition 1. If we have *J*_*p*_(**w**_*k*_, **c**_*k*_) ≤ *J*_*p*_(**w**_*k*+1_, **c**_*k*+1_) by fixing (α_*l*_, β_*l*_, γ_*l*_) = (α_*l, k*+1_, β_*l, k*+1_, γ_*l, k*+1_), the optimization problem (Eq. 15) will converge to a local optimum.

*Proof:* The convergence is proved as


(24) $$\begin{array}{l} \begin{array}{cc} J\left({\text{w}}_{k},{\text{c}}_{k},\alpha_{l,k},\beta_{l,k},\gamma_{l,k}\right)\\ \leq & J\left({\text{w}}_{k},{\text{c}}_{k},\alpha_{l,k+1},\beta_{l,k+1},\gamma_{l,k+1}\right)\\ \leq & J\left({\text{w}}_{k+1},{\text{c}}_{k+1},\alpha_{l,k+1},\beta_{l,k+1},\gamma_{l,k+1}\right) \end{array} \end{array}$$

in which the first inequality is guaranteed by the HQ mechanism (Ren and Yang, 2018), and the second inequality arises from the assumption of the present proposition.

One can observe that, to guarantee the convergence of Eq. (15), it is unnecessary to attain the strict maximum of Eq. (21) at each projector-step in Algorithm 1. On the contrary, so long as the updated projectors lead to a larger objective function *J*_*p*_ at each projector-step, Eq. (15) will converge to a local optimum. This reveals great convenience in practice, that one only needs a few SQP iterations for projector-step. One could finish the projector-step once confirming the increase on *J*_*p*_, thus accelerating the convergence.

### 4.3. Hyper-parameter determination

There exist five Gaussian kernel bandwidths σ_*x*_, σ_*y*_, σ_*r*_, σ_*p*_, and σ_*b*_, respectively, to be determined in practice. In the literature, an effective method to estimate a proper kernel bandwidth for probability density estimation, named as *Silverman's rule*, was proposed in Silverman (1986). Denoting the current set of errors as *E* with *L* observations, the kernel bandwidth is computed


(25) $$\begin{array}{l} \sigma^{2}=1.06\times \min\left\{\sigma_{E},\frac{R}{1.34}\right\}\times {\left(L\right)}^{-1/5} \end{array}$$

in which σ_*E*_ is the standard deviation of the *L* errors, and *R* denotes the interquartile range.

## 5. Experiments

In this section, we assessed the proposed PMCR algorithm on a synthetic dataset and a real ECoG dataset, respectively, comparing it with the existing PLSR methods. Specifically, we compared PMCR to the following methods: the conventional PLSR, the state-of-the-art regularized PLSR (RPLSR) (Foodeh et al., 2020) described in Eq. (7), and the rudimentary MCC-PLSR (Mou et al., 2018) described in Section 3.2. For a evenhanded comparison, each algorithm used an identical number of factors, which was selected by the conventional PLSR in five-fold cross-validation. The maximal number of factors was set as 100.

Considering the performance indicators for the evaluation, we used three typical measures in regression tasks: i) Pearson's correlation coefficient (*r*)


(26) $$\begin{array}{l} r=\frac{Cov\left(\hat{\text{Y}},\text{Y}\right)}{\sqrt{Var\left(\hat{\text{Y}}\right)Var\left(\text{Y}\right)}} \end{array}$$

where *Cov*(·, ·) and *Var*(·) denote the covariance and variance, respectively, and ii) root mean squared error (RMSE) which is computed by


(27) $$\begin{array}{l} \text{RMSE}=\sqrt{\frac{1}{L}\overset{L}{\underset{l=1}{\sum }}∥{\hat{\text{y}}}_{l}-{\text{y}}_{l}{∥}^{2}} \end{array}$$

in which ${\hat{\text{y}}}_{l}$ and **y**_*l*_ denote the *l*-th observations for the prediction $\hat{\text{Y}}$ and the target **Y**, respectively, and iii) mean absolute error (MAE) which represents the average *L*_1_-norm distance


(28) $$\begin{array}{l} \text{MAE}=\frac{1}{L}\overset{L}{\underset{l=1}{\sum }}∥{\hat{\text{y}}}_{l}-{\text{y}}_{l}∥ \end{array}$$

To compare the robustness between different algorithms, only contaminating the training samples by noises with isolating testing data from contamination is an extensively approved and implemented method in the literature, as advised in Zhu and Wu (2004). Accordingly, only the training sample would suffer the adverse contamination in the following experiments.

### 5.1. Synthetic dataset

#### 5.1.1. Dataset description

First, we considered an inter-correlated, high-dimensional, and noisy synthetic example, in which various PLSR methods were assessed with different levels of contamination. Randomly, we generated 300 i.i.d.^1^ latent variables **t**~*U*(0, 1) for training, and 300 i.i.d. latent variables **t**~*U*(0, 1) for testing, in which *U* denotes the uniform distribution, and the dimension of **t** was set as 20. We generated the hypothesis from the latent variable to the explanatory and response matrices then. Specifically, we randomly generated the transformation matrices with arbitrary values, which were subject to the standard normal distribution. The latent variables **t** were multiplied with a 20 × 500 transformation matrix, resulting in a 300 × 500 explanatory matrix for input. Similarly, we used a 20 × 3 transformation matrix to acquire a 300 × 3 response matrix for output. Accordingly, we predicted the multivariate responses from 500-dimensional explanatory variables with 300 training samples, and evaluated the prediction performance on the other 300 testing samples.

Considering the contamination for the synthetic dataset, we supposed the explanatory matrix to be contaminated, because the adverse noises mainly happen to the brain recordings, which are usually used as the explanatory in the BCI system. Therefore, a certain proportion (from 0 to 1.0 with a step 0.05) of training samples were randomly selected with equal probability, the inputs of which were then replaced by noises with large amplitude. For the distribution of the noise, we utilized a zero-mean Gaussian distribution with large standard deviation to imitate outliers, where 30, 100, and 300 were used, respectively.

#### 5.1.2. Results

**Figure 1:**
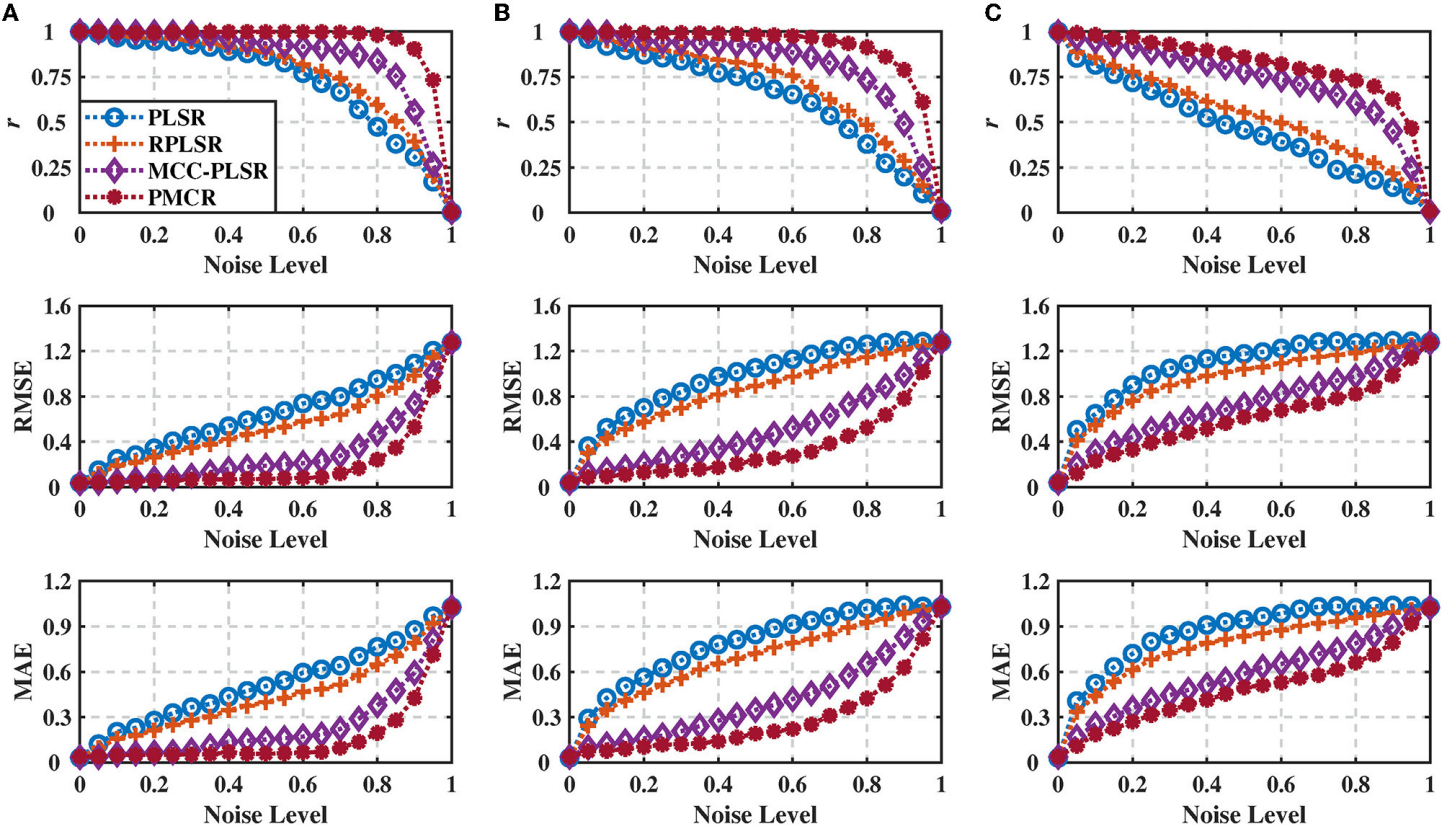
Regression performance indicators of the inter-correlated, high-dimensional, and contaminated synthetic dataset under different noise standard deviations with noise levels from 0 to 1.0. **(A)** Noise standard deviation = 30, **(B)** noise standard deviation = 100, and **(C)** noise standard deviation = 300. The performance indicators were acquired from 100 Monte-Carlo repetitive trials and averaged across three dimensions of the output. The proposed PMCR algorithm realized better performance than the existing PLSR algorithms consistently for *r*, RMSE, and MAE, in particular when the training set was contaminated considerably.

We evaluated the various PLSR methods with 100 Monte-Carlo repetitive trials, and present the results in Figure 1, where the results were averaged across three dimensions of the output. One could observe from Figure 1 that, for all the three different noise standard deviations, the proposed PMCR algorithm achieved superior prediction performance compared with the other existing methods consistently for *r*, RMSE, and MAE, respectively, in particular when the training set suffered considerable contamination.

**Figure 2:**
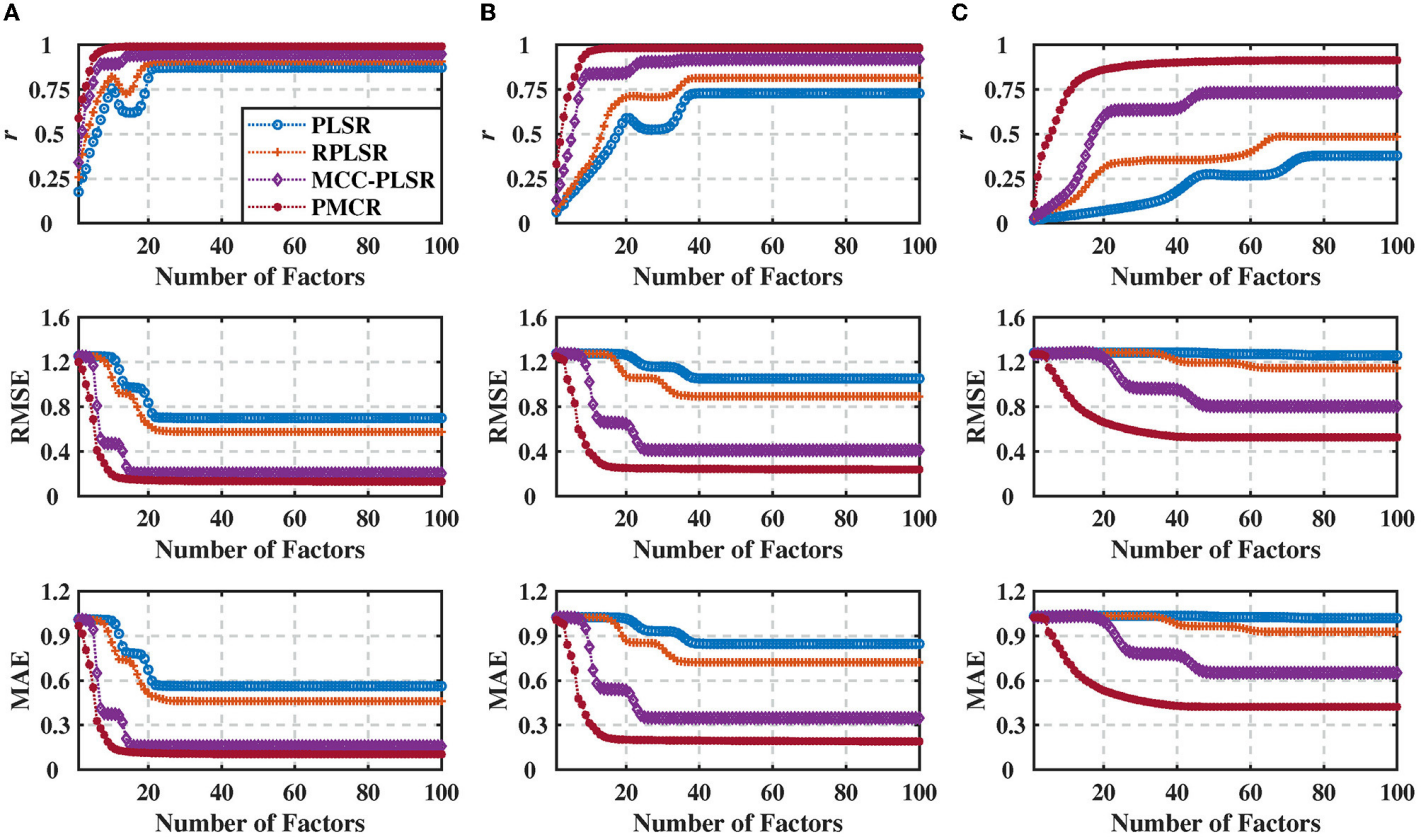
Regression performance indicators of the synthetic dataset with noise standard deviation being 100 under three different noise levels with the number of factors increasing from 1 to 100. **(A)** Noise level = 0.2, **(B)** noise level = 0.5, and **(C)** noise level = 0.8. The performance indicators were obtained from 100 repetitive trials and averaged across three dimensions of the output. The proposed PMCR algorithm not only acquired better prediction results than the other algorithms ultimately with the optimal number of factors, but also achieved admirable regression performance with a small number of factors.

The number of factors *S* plays a vital role in PLSR methods, representing the iteration numbers to decompose the input and output matrices. Since it usually causes a notable effect on the results, additionally, we evaluated the performance with respect to the number of factors for each method. To this end, we utilized the noise standard deviation 100 under three different noise levels, 0.2, 0.5, and 0.8, respectively. The prediction results for each method are presented in Figure 2 with 100 repetitive trials, with respect to the number of decomposition factors. One could perceive that, not only the proposed PMCR eventually achieved superior regression performance with the optimal number of factors, but also it realized rather commendable performance with a small number of factors. For example, when the noise level was equal to 0.5, the proposed PMCR achieved its optimal performance with no more than 20 factors. By comparison, for the other methods, when the number of factors was larger than 20, their performances remained promoting significantly. One can also observe a similar result in the other two noise levels. This suggests that, PMCR could abstract substantial information with a rather small number of factors from training samples in a noisy regression task.

### 5.2. ECoG dataset

To further demonstrate the superior robustness of the PMCR algorithm, we evaluated the various PLSR algorithms with the following practical brain decoding task. In this subsection, we used the publicly available Neurotycho ECoG dataset^2^ which was initially proposed in Shimoda et al. (2012).

#### 5.2.1. Dataset description

**Figure 3:**
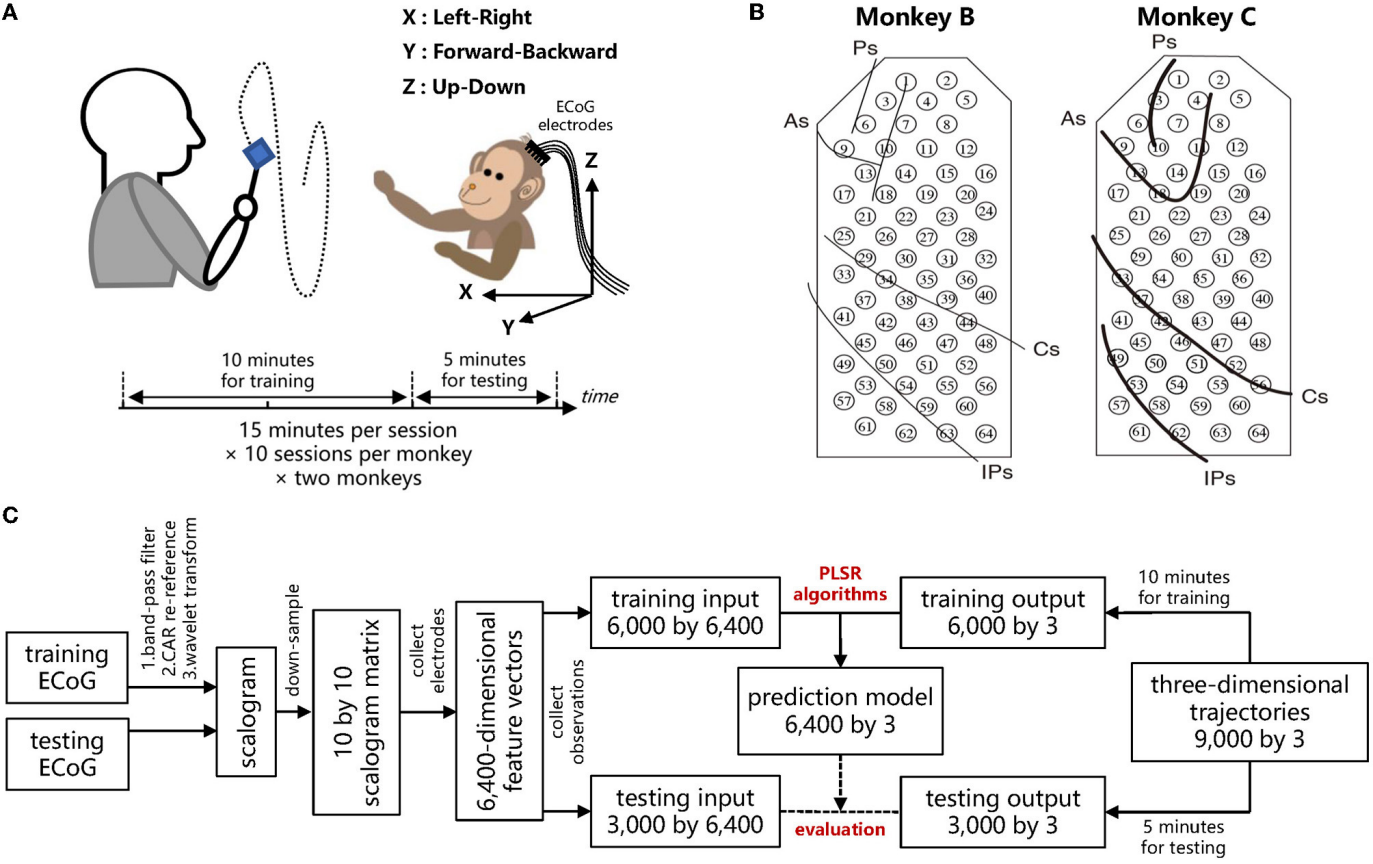
Experimental protocol of the Neurotycho ECoG dataset and decoding paradigm to evaluate the robustness of the different PLSR algorithms. **(A)** The macaque retrieved foods in a three-dimensional random location, during which the body-centered coordinates of the right wrists and the ECoG signals were recorded simultaneously. **(B)** Both Monkey B and C were implanted with 64-channel epidural ECoG electrodes on the contralateral (left) hemisphere, overlaying the regions from the prefrontal cortex to the parietal cortex. Ps: principal sulcus, As: arcuate sulcus, Cs: central sulcus, IPs: intraparietal sulcus. **(A, B)** Were reproduced from Shimoda et al. (2012), which provides the details of this public dataset. **(C)** Decoding diagram from ECoG signals to three-dimensional trajectories. The training ECoG signals are contaminated to assess the robustness of different algorithms.

Two Japanese macaques, denoted by Monkey B and C, respectively, were commanded to track foods with the right hands, during which the continuous three-dimensional trajectories of right hands with a sampling rate of 120 Hz were recorded by an optical motion capture instrument. For both Monkey B and C, ten recording sessions were performed, where each recording session lasted 15 minutes. The two macaques were in advance implanted with customized 64-channel ECoG electrodes on the contralateral (left) hemisphere, which covered the regions from the prefrontal cortex to the parietal cortex. ECoG signals were recorded simultaneously during each session with a sampling rate of 1,000 Hz. In accordance with Shimoda et al. (2012), for each recording session, the data of the first ten minutes was used to train a prediction model, while the data of the remaining five minutes was used to evaluate the prediction performance of the trained model. The schemes of the experiments and ECoG electrodes are shown in Figures 3A, B, respectively.

#### 5.2.2. Decoding paradigm

For the feature extraction, we used an identical offline decoding paradigm as in Shimoda et al. (2012). Initially, ECoG signals were preprocessed with a tenth-order Butterworth bandpass filter with cutoff frequencies from 1 to 400 Hz, and then re-referenced by the common average referencing (CAR) method. The three-dimensional trajectories of the right wrist were down-sampled to 10 Hz, thus, leading to 9,000 samples in one session (10 Hz × 60 sec × 15 min). The three-dimensional position of time *t* was predicted from the ECoG signals during the previous one second. To extract the features of ECoG signals, we utilized the time-frequency representation. For the time *t*, the ECoG signals at each electrode from *t*- 1.1 s to *t* were processed by Morlet wavelet transformation. Ten center frequencies ranging from 10 to 120 Hz with equal spacing on the logarithmic scale were considered for the wavelet transformation, overlaying the frequency bands which are most relevant to motion tasks (Shimoda et al., 2012). The time- frequency scalogram was then resampled at ten temporal lags with a 0.1 s gap (*t*- 1 s, *t*- 0.9 s,..., *t*- 0.1 s). Thus, the input of each sample exhibited a 6,400-dimensional vector (64 channels × 10 frequencies × 10 temporal lags), and the output was the three-dimensional position of the right hand. Hence, we trained a regression model with 6,000 samples (the first ten minutes) to predict the three-dimensional output from the 6,400-dimensional input, and evaluated the algorithms with other 3,000 testing samples (the remaining five minutes). The illustrative diagrams for ECoG decoding are summarized in Figure 3C.

#### 5.2.3. Contamination

To evaluate the robustness of different algorithms in the practical ECoG decoding task, the ECoG signals were artificially contaminated by outlier to simulate the detrimental artifact. To be specific, we stochastically selected three certain proportions, 0 (no contamination), 10^−3^, and 10^−2^, of the training ECoG samplings and corrupted them with outliers which were subject to the zero-mean Gaussian distribution with the variance 50 times that of the signals for the corresponding channel. As stated in Ball et al. (2009a), the blink-related artifacts were remarkably found in ECoG signal that exhibited much larger amplitudes than a normal ECoG recording. Hence, we used the above-mentioned approach to artificially generate adverse artifacts, so as to contaminate the ECoG signals. This method has been widely utilized in the literature to deteriorate the brain signals for evaluating the robustness of different algorithms (Wang et al., 2011; Chen et al., 2018).

**Figure 4:**
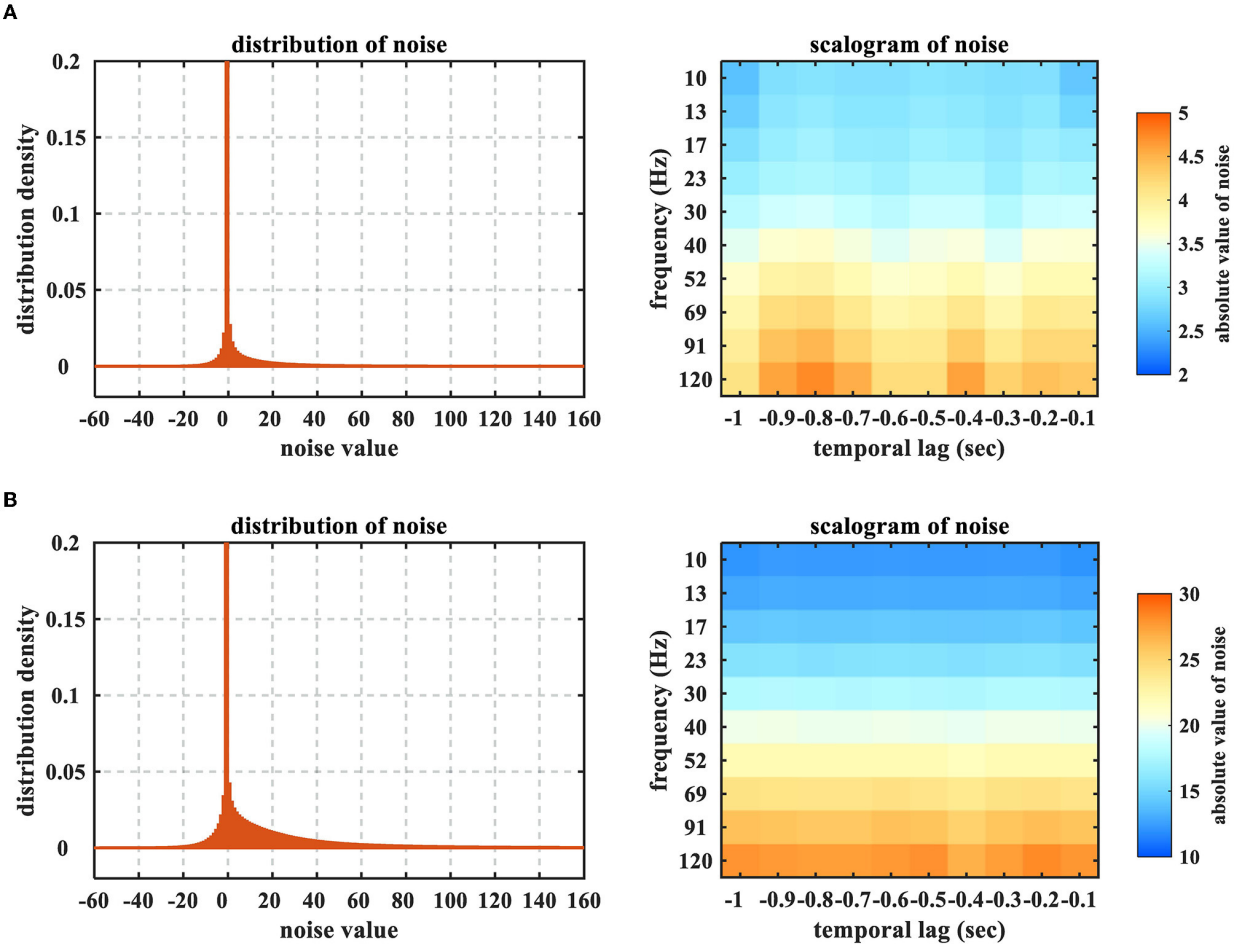
Distributions and scalograms of the time-frequency feature noises resulting from the ECoG sampling contamination. **(A)** Noise level = 10^−3^ (the deteriorated proportion of training set = 0.6645 ± 0.0089), **(B)** Noise level = 10^−2^ (the deteriorated proportion of training set ≈1). The time-frequency feature noises were calculated by subtracting the training datasets which were obtained from acoustic and contaminated ECoG signals, respectively. The distributions were averaged by 20 sessions of Monkey B and C, while the scalograms were averaged across all electrodes. The peaks of distributions are truncated to emphasize the heavy-tailed characteristic.

Note that, for this ECoG dataset, the ‘Noise Level' signifies the ratio of the contaminated ECoG samplings in the entirety which is different from the ratio of the deteriorated samples in the 6,000 training samples. The ratio of the affected training samples can be evidently larger than the indicated noise level, since one contaminated ECoG sampling could deteriorate several time windows in feature extraction. For example, when the noise level is denoted as 10^−3^, the deteriorated proportion of the training set is (0.6645 ± 0.0089). Furthermore, we illustrate how the noise would influence the time-frequency feature in Figure 4. One could obviously perceive the heavy-tailed characteristic on the feature noises, which is in particular intractable for the least square criterion. In addition, the effects of high-frequency band are more prominent, due to the property of impulsive noise.

#### 5.2.4. Spatio-spectro-temporal pattern

Studying how the spatio-spectro-temporal weights in the regression model contribute to the entirety can help investigate the neurophysiological pattern. The element of the trained prediction model **H** can be denoted by *h*_ch, freq, temp_, which corresponds to the ECoG electrode “ch,” the frequency “freq,” and the temporal lag “temp.” Thus, one could calculate the spatio-spectro-temporal contributions by the ratio between the summation of absolute values of each domain and the summation of absolute values of the entire model


(29) $$\begin{array}{l} W_{c}\left(\text{ch}\right)=\frac{\sum_{\text{freq}}\sum_{\text{temp}}|h_{\text{ch},\text{freq},\text{temp}}|}{\sum_{\text{ch}}\sum_{\text{freq}}\sum_{\text{temp}}|h_{\text{ch},\text{freq},\text{temp}}|} \end{array}$$

(30) $$\begin{array}{l} W_{f}\left(\text{freq}\right)=\frac{\sum_{\text{ch}}\sum_{\text{temp}}|h_{\text{ch},\text{freq},\text{temp}}|}{\sum_{\text{ch}}\sum_{\text{freq}}\sum_{\text{temp}}|h_{\text{ch},\text{freq},\text{temp}}|} \end{array}$$

(31) $$\begin{array}{l} W_{t}\left(\text{temp}\right)=\frac{\sum_{\text{ch}}\sum_{\text{freq}}|h_{\text{ch},\text{freq},\text{temp}}|}{\sum_{\text{ch}}\sum_{\text{freq}}\sum_{\text{temp}}|h_{\text{ch},\text{freq},\text{temp}}|} \end{array}$$

where *W*_*c*_(ch), *W*_*f*_(freq), and *W*_*t*_(temp) denote the contributions of the ECoG electrode “ch,” the frequency “freq,” and the temporal lag “temp,” respectively.

#### 5.2.5. Results

First, we assessed the different algorithms with the uncontaminated ECoG signals. Accordingly, when the noise level was zero, the average performance indicators were obtained by the acoustic 20 sessions (Monkey B and C). Then we contaminated each session with 5 repetitive trials. Hence, for every noise level, each algorithm was evaluated for 100 times (20 sessions × 5 repetitive trials). In Table 2, we present the performance indicators for each algorithm with the noise levels 0, 10^−3^, and 10^−2^, respectively. In each row of a specific condition, the optimal result is marked in bold. Moreover, the other results are marked with (*) if there exists statistically significant difference between the current result and the optimal result under each condition. One observes in Table 2 that, the proposed PMCR realized the optimal prediction results consistently, except for the Y-axis under noise level 0. In most cases, PMCR outperformed the other methods with statistically significant difference. One can observe that, when the noise level was 0, PMCR achieved better results than the other algorithms for X-axis and Z-axis. One major reason is, in the acoustic sessions, the motion-related artifacts have been considerably found in the ECoG signals (Shimoda et al., 2012), which further demonstrates the necessity of utilizing PMCR in the practical ECoG decoding tasks.

**Table 2:** Performance indicators of each algorithm on the Neurotycho ECoG dataset under three noise levels 0, 10^−3^, and 10^−2^, respectively.

**X-position**						
**Algorithm**			**PLSR**	**RPLSR**	**MCC-PLSR**	**PMCR**
makecell	0	*r*	0.4378 ± 0.0933^*^	0.4550 ± 0.0925^*^	0.4598 ± 0.0942^*^	**0.4679** **±** **0.0947**
		RMSE	0.9287 ± 0.0810^*^	0.9037 ± 0.0653^*^	0.8954 ± 0.0809^*^	**0.8835** **±** **0.0786**
		MAE	0.7026 ± 0.0640^*^	0.6872 ± 0.0530^*^	0.6749 ± 0.0628^*^	**0.6658** **±** **0.0651**
	10^−3^	*r*	0.3334 ± 0.1165^*^	0.3558 ± 0.1132^*^	0.3684 ± 0.1127^*^	**0.3873** **±** **0.1274**
		RMSE	0.9729 ± 0.0652^*^	0.9543 ± 0.0648^*^	0.9397 ± 0.0728^*^	**0.9276** **±** **0.0705**
		MAE	0.7291 ± 0.0756^*^	0.7174 ± 0.0689^*^	0.7092 ± 0.0786^*^	**0.6987** **±** **0.0759**
	10^−2^	*r*	0.1524 ± 0.1399^*^	0.1713 ± 0.1353^*^	0.1926 ± 0.1342^*^	**0.2238** **±** **0.1382**
		RMSE	1.0249 ± 0.1105^*^	1.0022 ± 0.1097^*^	0.9845 ± 0.1129^*^	**0.9681** **±** **0.1094**
		MAE	0.7655 ± 0.1428^*^	0.7485 ± 0.1383^*^	0.7396 ± 0.1392^*^	**0.7246** **±** **0.1397**
				**Y-position**		
**Algorithm**			**PLSR**	**RPLSR**	**MCC-PLSR**	**PMCR**
makecell	0	*r*	0.5426 ± 0.1019^*^	**0.5582** **±** **0.1026**	0.5547 ± 0.1017	0.5549 ± 0.1022
		RMSE	0.8483 ± 0.0969^*^	**0.8198** **±** **0.0951**	0.8246 ± 0.0948	0.8233 ± 0.0952
		MAE	0.6487 ± 0.0762^*^	**0.6304** **±** **0.0796**	0.6362 ± 0.0744	0.6358 ± 0.0759
	10^−3^	*r*	0.4114 ± 0.1309 ^*^	0.4284 ± 0.1285^*^	0.4425 ± 0.1302^*^	**0.4602** **±** **0.1296**
		RMSE	0.9188 ± 0.0963 ^*^	0.8962 ± 0.0958^*^	0.8795 ± 0.0979^*^	**0.8608** **±** **0.1002**
		MAE	0.6960 ± 0.1007^*^	0.6849 ± 0.1014^*^	0.6631 ± 0.0983^*^	**0.6539** **±** **0.1021**
	10^−2^	*r*	0.2084 ± 0.1514^*^	0.2206 ± 0.1489^*^	0.2593 ± 0.1502^*^	**0.2723** **±** **0.1537**
		RMSE	0.9781 ± 0.1143^*^	0.9542 ± 0.1117^*^	0.9306 ± 0.1159	**0.9294** **±** **0.1146**
		MAE	0.7354 ± 0.1028^*^	0.7173 ± 0.1077^*^	0.7086 ± 0.1105	**0.7043** **±** **0.1042**
				**Z-position**		
**Algorithm**			**PLSR**	**RPLSR**	**MCC-PLSR**	**PMCR**
makecell	0	*r*	0.6320 ± 0.0324^*^	0.6395 ± 0.0328^*^	0.6482 ± 0.0359	**0.6504** **±** **0.0372**
		RMSE	0.7968 ± 0.0281^*^	0.7814 ± 0.0293^*^	0.7747 ± 0.0296^*^	**0.7628** **±** **0.0275**
		MAE	0.6181 ± 0.0222^*^	0.6102 ± 0.0280^*^	0.6055 ± 0.0241	**0.5989** **±** **0.0265**
	10^−3^	*r*	0.4875 ± 0.0708^*^	0.4935 ± 0.0701^*^	0.5158 ± 0.0857^*^	**0.5259** **±** **0.0814**
		RMSE	0.9272 ± 0.0712^*^	0.9129 ± 0.0682^*^	0.8958 ± 0.0742^*^	**0.8834** **±** **0.0738**
		MAE	0.6932 ± 0.0800^*^	0.6894 ± 0.0814^*^	0.6804 ± 0.0852^*^	**0.6645** **±** **0.0782**
	10^−2^	*r*	0.2399 ± 0.1185^*^	0.2456 ± 0.1173^*^	0.2615 ± 0.1148^*^	**0.2803** **±** **0.1186**
		RMSE	1.0168 ± 0.0804^*^	0.9917 ± 0.0785^*^	0.9605 ± 0.0842^*^	**0.9485** **±** **0.0809**
		MAE	0.7532 ± 0.0883^*^	0.7429 ± 0.0892^*^	0.7208 ± 0.0893	**0.7146** **±** **0.0887**

The results are given in mean ± deviation, where the optimal results under each condition are marked in bold. The proposed PMCR realized the optimal results consistently, except for the Y-position under the noise level 0. For each result, ^*^is marked if there exists statistically significant difference between the indicated one and the optimal result in the corresponding condition, according to a paired *t*-test (*p* < 0.05).

**Figure 5:**
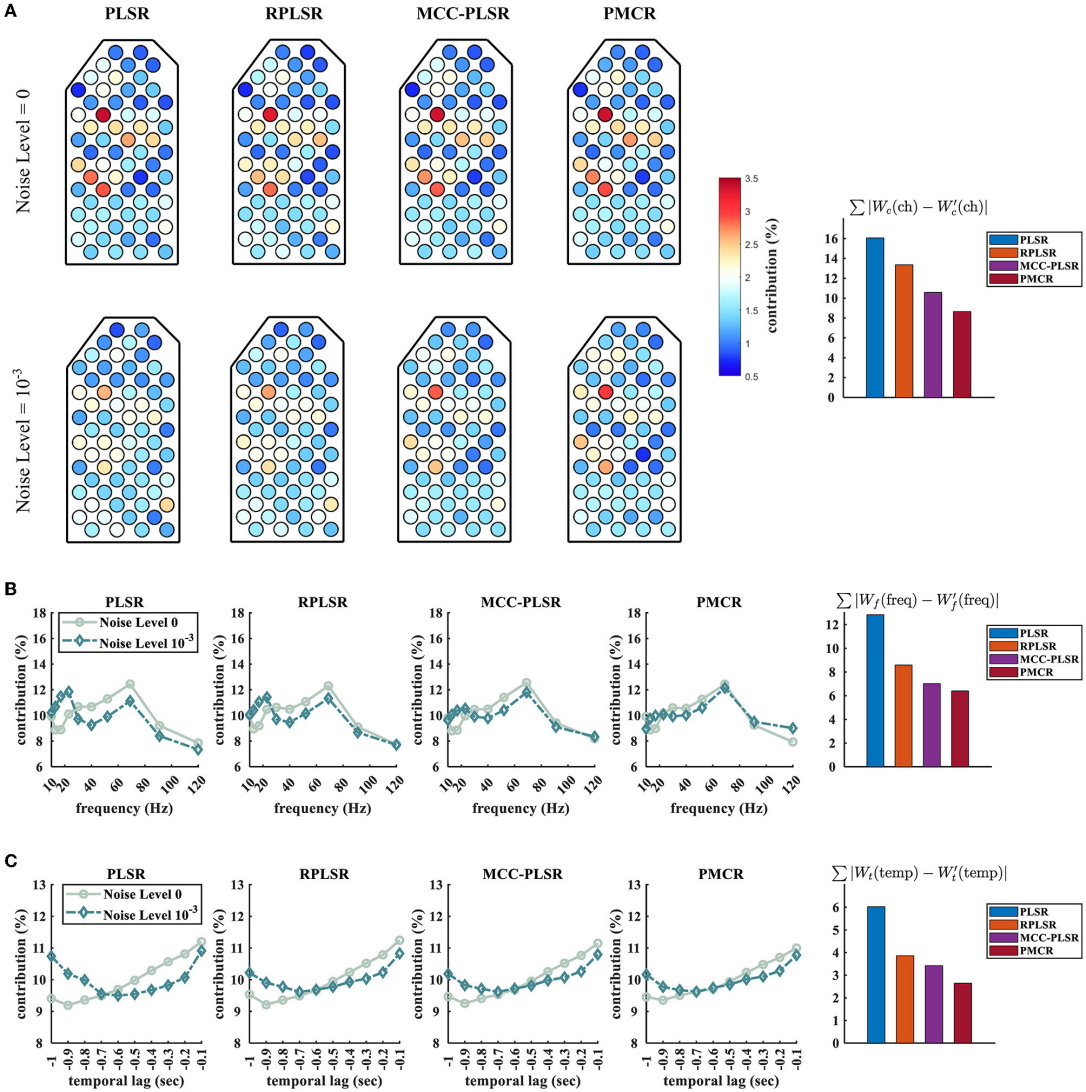
Spatio-spectro-temporal contributions of the prediction model for Monkey B's Z-position under noise levels 0 and 10^−3^. **(A)** Spatial patterns, **(B)** spectral patterns, and **(C)** temporal patterns. For each domain, the quantitative deterioration is calculated by the absolute value summation of the difference between the original and the deteriorated patterns. The original patterns *W*_*c*_(ch), *W*_*f*_(freq), and *W*_*t*_(temp) were averaged across the 10 acoustic sessions of Monkey B, while the deteriorated patterns $W_{c}^{\prime }\left(\text{ch}\right)$, $W_{f}^{\prime }\left(\text{freq}\right)$, and $W_{t}^{\prime }\left(\text{temp}\right)$ were averaged across 50 trials (10 sessions of Monkey B × 5 repetitive trials). The proposed PMCR achieved the minimal deterioration for each domain.

Furthermore, we studied how the neurophysiological patterns for different algorithms were influenced by the sampling noises. We show the differences between the spatial, the spectral, and the temporal contributions which were acquired from the acoustic and the contaminated sessions (under the noise level 10^−3^), respectively, in Figure 5. The regression model concerning Monkey B's Z-position was used here. We also quantified the effects by computing the summation of the absolute values of the difference between the patterns that were attained from the acoustic and the contaminated sessions, respectively. To be specific, we illustrate $\sum |W_{c}\left(\text{ch}\right)-W_{c}^{\prime }\left(\text{ch}\right)|$, $\sum |W_{f}\left(\text{freq}\right)-W_{f}^{\prime }\left(\text{freq}\right)|$, and $\sum |W_{t}\left(\text{temp}\right)-W_{t}^{\prime }\left(\text{temp}\right)|$ for the spatial, the spectral, and the temporal patterns, respectively. *W*_*c*_(ch), *W*_*f*_(freq), and *W*_*t*_(temp) were obtained by the acoustic sessions, while $W_{c}^{\prime }\left(\text{ch}\right)$, $W_{f}^{\prime }\left(\text{freq}\right)$, and $W_{t}^{\prime }\left(\text{temp}\right)$ were obtained from the contaminated sessions. One can observe from Figure 5 that, the proposed PMCR algorithm realized the minimal deterioration for the pattern of each domain. This further demonstrates the robustness of PMCR in noisy ECoG decoding tasks.

## 6. Discussion

### 6.1. Proposed method

**Figure 6:**
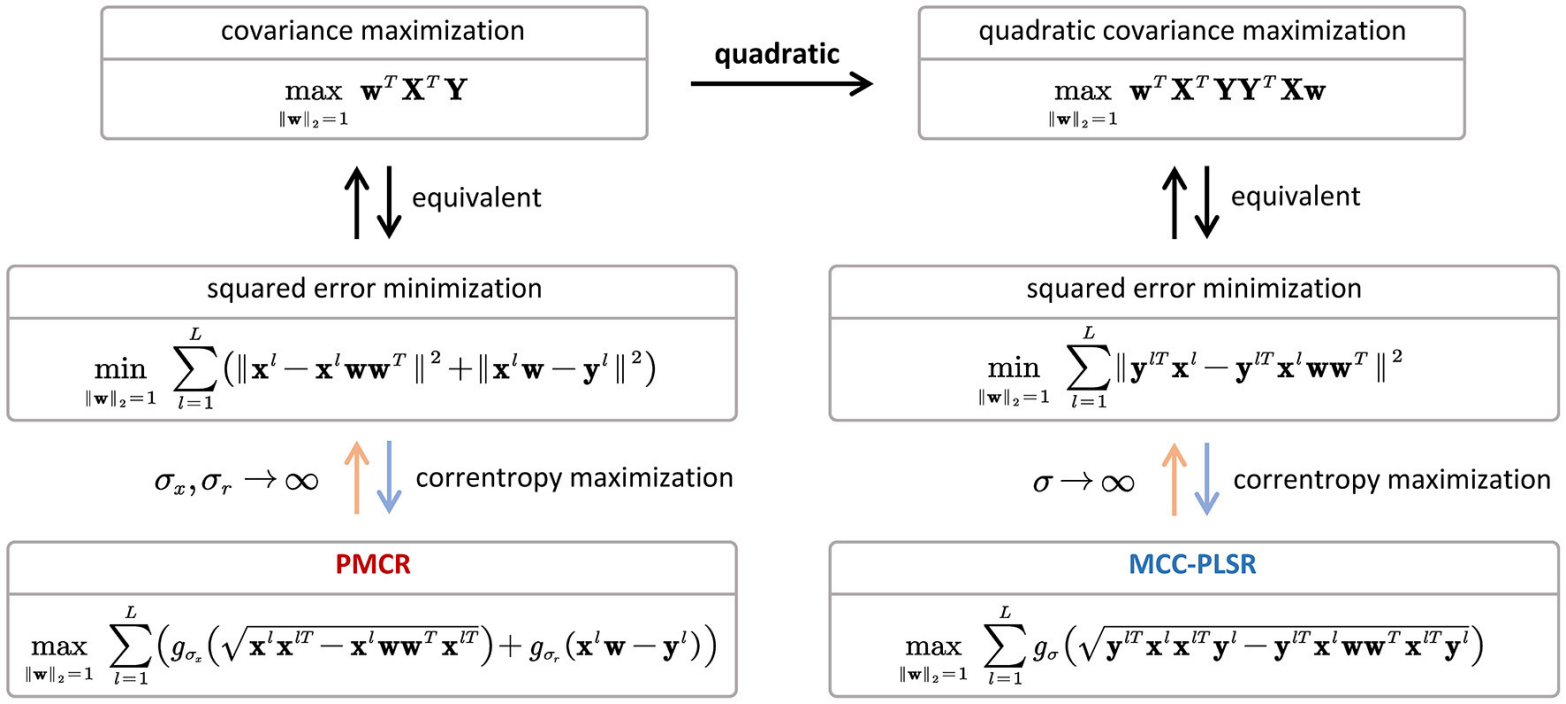
The connection between PMCR and MCC-PLSR for a univariate response.

In the present study, we aimed to propose a new robust version for PLSR using the MCC framework, which is named as PMCR. Similarly as the existing PLSR methods, the proposed PMCR decomposes the explanatory matrix (input) and the response matrix (output) iteratively for *S* decomposition factors. The crucial differences of the proposed PMCR are stated in what follows. First, the objective function regarding the projectors **w**_*s*_ and **c**_*s*_ in Eq. (15) could be considered as a generalized formulation of the conventional PLSR (Eq. 6), and would be closely related to the calculation in MCC-PLSR (Eq. 12) under specific conditions. As has been proved in Liu et al. (2007), maximizing the correntropy between two variables, when the kernel bandwidth tends to infinity, is equal to minimizing their quadratic Euclidean distance. Hence, if we suppose σ_*x*_, σ_*y*_, σ_*r*_ → ∞, the projector calculation of PMCR will degenerate to the conventional PLSR. Then, we consider the differences between MCC-PLSR and the proposed PMCR. For a univariate response, the projector **c** for dimensionality reduction regarding the response could be ignored. Thus, we can rewrite the dimensionality reduction in PMCR (Eq. 15) as


(32) $$\begin{array}{l} \underset{∥\text{w}{∥}_{2}=1}{\max}\overset{L}{\underset{l=1}{\sum }}\left(g_{\sigma_{x}}\left(\sqrt{{\text{x}}^{l}{\text{x}}^{lT}-{\text{x}}^{l}\text{w}{\text{w}}^{T}{\text{x}}^{lT}}\right)+g_{\sigma_{r}}\left({\text{x}}^{l}\text{w}-{\text{y}}^{l}\right)\right) \end{array}$$

which could be regarded as a generalized form for the quadratic error minimization (Liu et al., 2007).


(33) $$\begin{array}{l} \underset{∥\text{w}{∥}_{2}=1}{\min}\overset{L}{\underset{l=1}{\sum }}\left(∥{\text{x}}^{l}-{\text{x}}^{l}\text{w}{\text{w}}^{T}{∥}^{2}+∥{\text{x}}^{l}\text{w}-{\text{y}}^{l}{∥}^{2}\right)\Leftrightarrow \underset{∥\text{w}{∥}_{2}=1}{\max}\;{\text{w}}^{T}{\text{X}}^{T}\text{Y} \end{array}$$

which is essentially equal to the conventional PLSR for univariate output. By comparison, MCC-PLSR adopts the MCC framework for the quadratic covariance (Eq. 11), which can be written as Mou et al. (2018)


(34) $$\begin{array}{l} \underset{∥\text{w}{∥}_{2}=1}{\min}\overset{L}{\underset{l=1}{\sum }}∥{\text{y}}^{lT}{\text{x}}^{l}-{\text{y}}^{lT}{\text{x}}^{l}\text{w}{\text{w}}^{T}{∥}^{2}\Leftrightarrow \underset{∥\text{w}{∥}_{2}=1}{\max}{\text{w}}^{T}{\text{X}}^{T}\text{Y}{\text{Y}}^{T}\text{X}\text{w} \end{array}$$

which is the special case of MCC-PLSR when the kernel bandwidth in Eq. (12) tends to infinity. Thus, the connection between PMCR (Eq. 15) and MCC-PLSR (Eq. 12) could be illustrated as in Figure 6. One can observe that, the starting points of PMCR and MCC-PLSR are different. The proposed PMCR begins from the original covariance maximization, whereas MCC-PLSR was proposed from the quadratic covariance. Therefore, we argue that our proposed PMCR is a more rational robust implementation for PLSR. Moreover, note that we give the above discussion under the premise of a univariate output, which is only a special case of degradation for our proposed PMCR. One the other hand, considering the calculations of the loading vector **p**_*s*_ and the regression coefficient *b*_*s*_, the proposed PMCR employs the MCC (Eq. 16, 17), whereas the conventional PLSR and MCC-PLSR utilize the least square criterion. As mentioned above, Eqs. (16, 17) can be also regarded as generalized forms of square error minimization. In summary, the proposed PMCR is more generalized than the conventional PLSR and MCC-PLSR.

In addition, we would like to discuss the advantages and disadvantages of the proposed PMCR algorithm. The essential benefit of utilizing the PMCR algorithm in a noisy ECoG decoding task is the conspicuous robustness with respect to the noises, which was demonstrated with extensive experiments in Section 5. Further, mathematically, the proposed PMCR algorithm is more generalized than the conventional PLSR and MCC-PLSR. As was mentioned above, the conventional PLSR and MCC-PLSR could be regarded as special cases of the proposed PMCR under specific conditions concerning the kernel bandwidths. In particular, compared with MCC-PLSR, the proposed PMCR takes into account the dimensionality reduction for the response matrix. As a result, PMCR could realize better prediction performance for multivariate response. Moreover, PMCR could be further implemented with regularization techniques and extended to the multi-way scenario, which would be discussed in the following subsections. However, PMCR might suffer the performance degradation resulting from inadequate kernel bandwidths that are calculated by the *Silverman's rule* (Eq. 25). Although the experimental results in this study verified empirically that, the proposed PMCR could perform efficiently with the kernel bandwidths acquired by Eq. (25), it may be difficult to guarantee that the *Silverman's rule* can always provide adequate kernel bandwidths. Hence, we would like to investigate a better way to determine the kernel bandwidths with solid theoretical guarantees in our future works. In addition, our proposed PMCR is effective to deal with outliers, while it may be inadequate for multi-modal-distributed noise because MCC utilizes only one kernel function for each reconstruction error. To address this issue, it is promising to use *minimum error entropy* (MEE) to reformulate PLSR, another popular learning criterion in ITL (Principe, 2010). MEE employs multiple kernel functions for each reconstruction error, so that it can realize satisfactory robustness with respect to multi-modal-distributed noise, which has realized robust neural decoding algorithms (Chen et al., 2018; Li et al., 2021).

### 6.2. PMCR with regularization

One should additionally note that, the PMCR was proposed by reformulating the conventional PLSR algorithm with using the robust MCC, instead of the mediocre least square criterion. Hence, the proposed PMCR exhibits the supplementary potential for further performance improvements with regularization techniques, as well as in the existing regularized PLSR methods. For example, *L*_1_-regularization could be utilized in Eq. (15) to encourage sparse and robust projectors. In addition, if one requires better smoothness on the predicted output, polynomial or Sobolev-norm penalization could be utilized in PMCR. Moreover, *L*_2_-regularization could be utilized for Eq. (17) to decrease the over-fitting risk considering the regression scalar *b*_*s*_, similarly as Eq. (7) (Foodeh et al., 2020). In the literature, MCC-based algorithms with regularization have been widely investigated. For instance, a robust version of sparse representation classifier (SRC) for face recognition was developed by employing *L*_1_-regularization on the MCC-based SRC objective function (He et al., 2010).

### 6.3. Extension to multi-way application

The multi-way PLSR establishes the regression relationship between tensor variables with dimensionality reduction by tensor factorization technique. In the literature, the multi-way PLSR was usually reported to achieve better decoding capability than the generic PLSR algorithm in the brain decoding task, where the spatio-spectro-temporal feature is organized with the tensor form. Essentially, the multi-way PLSR decomposes the input and output under the least square criterion by minimizing the Frobenius-norm (Kolda and Bader, 2009). Therefore, the multi-way PLSR is prone to the performance deterioration caused by noises as well.

The proposed PMCR method treats the regression problem of matrix, i.e. two-way variable. Extending the PMCR algorithm to multi-way application could probably improve the prediction performance further, which would be investigated in our future works. Promisingly, MCC has been demonstrated effective for tensor variable analysis in a recent study (Zhang et al., 2016).

## 7. Conclusion

This paper proposed a new robust variant for the PLSR algorithm by reformulating the non-robust least square criterion with the sophisticated MCC framework. The proposed robust objective functions can be effectively optimized by half-quadratic and fixed-point optimization methods. Extensive experimental results with the synthetic dataset and Neurotycho epidural ECoG dataset demonstrate that, the proposed PMCR can outperform the existing PLSR algorithms, revealing promising robustness for high-dimensional and noisy ECoG decoding tasks.

## Data availability statement

The original contributions presented in the study are included in the article/supplementary material, further inquiries can be directed to the corresponding author.

## Author contributions

YL and BC contributed to conceptualization of the study and developed the algorithm derivation. YL and GW operated the experiments and analyzed the results. YL wrote the first draft of the manuscript. NY and YK directed the study and guided the manuscript. All authors contributed to the article and approved the submitted version.

## Footnotes

^1^Independent and identically distributed.

^2^Available online at http://neurotycho.org/epidural-ecog-food-tracking-task.
